# Volatile-Mediated Induced and Passively Acquired Resistance in Sagebrush (*Artemisia tridentata*)

**DOI:** 10.1007/s10886-022-01378-y

**Published:** 2022-08-19

**Authors:** Patrick Grof-Tisza, Natasja Kruizenga, Arja I. Tervahauta, James D. Blande

**Affiliations:** 1 Department of Environmental and Biological Sciences, University of Eastern Finland, Kuopio, Finland; 2 Institute of Biology, University of Neuchâtel, Neuchâtel, Switzerland; 3 HAS University of Applied Sciences, Onderwijsboulevard, Netherlands

**Keywords:** Associational resistance, chemotype, induced resistance, kin selection, volatile signaling

## Abstract

Plants produce a diversity of secondary metabolites including volatile organic compounds. Some species show discrete variation in these volatile compounds such that individuals within a population can be grouped into distinct chemotypes. A few studies reported that volatile-mediated induced resistance is more effective between plants belonging to the same chemotype and that chemotypes are heritable. The authors concluded that the ability of plants to differentially respond to cues from related individuals that share the same chemotype is a form of kin recognition. These studies assumed plants were actively responding but did not test the mechanism of resistance. A similar result was possible through the passive adsorption and reemission of repellent or toxic VOCs by plants exposed to damage-induced plant volatiles (DIPVs). Here we conducted exposure experiments with five chemotypes of sagebrush in growth chambers; undamaged receiver plants were exposed to either filtered air or DIPVs from mechanically wounded branches. Receiver plants exposed to DIPVs experienced less herbivore damage, which was correlated with increased expression of genes involved in plant defense as well as increased emission of repellent VOCs. Plants belonging to two of the five chemotypes exhibited stronger resistance when exposed to DIPVs from plants of the same chemotypes compared to when DIPVs were from plants of a different chemotype. Moreover, some plants passively absorbed DIPVs and reemitted them, potentially conferring associational resistance. These findings support previous work demonstrating that sagebrush plants actively responded to alarm cues and that the strength of their response was dependent on the chemotypes of the plants involved. This study provides further support for kin recognition in plants but also identified volatile-mediated associational resistance as a passively acquired additional defense mechanism in sagebrush.

**Supplementary Information:**

The online version contains supplementary material available at 10.1007/s10886-022-01378-y.

## Introduction

Despite their lack of conspicuous organs dedicated to perception, plants can sense the surrounding community including herbivores (Wu and Baldwin 2009; Appel and Cocroft 2014) and other plants (Callaway 2002). Independent of pollen self-incompatibility mechanisms (Charlesworth et al. 2005), a growing number of studies have shown that plants can distinguish whether neighbouring plants are kin or strangers (Dudley and File 2007; Murphy and Dudley 2009; Karban et al. 2013; Crepy and Casal 2015). Upon recognizing kin, a plant may act altruistically towards its relatives, such as decreasing competition for shared resources (Dudley and File 2007; Crepy and Casal 2015). These altruistic behaviours are evolutionarily favourable when they increase the inclusive fitness of the actor beyond any derived costs (Hamilton 1964).

Plants produce a diversity of secondary metabolites, including volatile organic compounds (VOCs) that are emitted into the atmosphere (Knudsen and Gershenzon 2006). The identity and concentrations of VOCs comprising a plant’s total volatile emission bouquet vary substantially depending on several factors, including life-stage, abiotic conditions, and biotic and abiotic stress (Dudareva et al. 2006, 2013). For example, the blend of VOCs emitted from herbivore-damaged tissue, is compositionally different from undamaged tissue (Hare 2011). These herbivore-induced plant volatiles (HIPVs) carry reliable information such as the identity of the damaged tissue and the attacking insect. Consequently, HIPVs are used by many interacting organisms; they can attract (Dicke and van Loon 2000) or can repel (De Moraes et al. 2001; Khan et al. 2008) other herbivores, and natural enemies often use these cues to locate their prey (Turlings and Tumlinson 1992; Kessler and Baldwin 2001). Damage-induced plant volatiles also play a role in plant signalling. They can serve as alarm cues for undamaged tissue of the same plant, activating defensive pathways leading to greater resistance to herbivores and a reduction of damage in subsequent attacks (Karban et al. 2006; Kost and Heil 2006; Li and Blande 2017). Neighbouring plants can eavesdrop on within-plant signalling and increase their own resistance without experiencing damage themselves (Heil and Karban 2010; Karban 2015).

Despite finding evidence for volatile-mediated plant-to-plant signalling in more than 50 species, the specificity of the cue needed to initiate a response is poorly understood (Karban et al. 2014b; Karban 2015). It is unclear why some plants can perceive and respond to volatile cues from different species, while others cannot (Karban et al. 2000, 2003; Glinwood et al. 2004; Heil and Karban 2010). Recent work demonstrated that plants could discriminate between volatile cues from kin or strangers (Karban et al. 2013; Karban et al. 2014a; Hussain et al. 2019). Chemical analysis revealed that the volatile emissions of these plants exhibited discrete variation and were subsequently classified into non-plastic chemotypes based on compounds that dominated the blends. Kin generally shared the same chemotype, while strangers were generally of a different chemotype. Additional work revealed that chemotypes are often heritable (Karban et al. 2014a; György et al. 2020). These findings suggest that the ability of plants to recognize kin and respond to emitted cues is in part based on the chemotypes of the interacting emitting and receiving plants in species that exhibit chemotypic variation of volatile emissions. This work led to formulation of the kin selection hypothesis (KSH) (Karban et al. 2013) which posits that selection should favour the privatization of volatile alarm cues such that only closely related individuals can perceive and respond to their damaged relatives. Under the KSH, plants receiving VOC cues (hereafter ‘receivers’) directly benefit through induced resistance while plants that emit VOCs (hereafter ‘emitters’) indirectly benefit through increasing the fitness of their relatives and thereby themselves (i.e., inclusive fitness). However, this hypothesis and more generally, the specificity of alarm cues needed to elicit a response, has seen limited testing [but see (Kalske et al. 2019; Grof-Tisza et al. 2021)].

The KSH assumes that receiver plants perceive volatile cues from related plants and actively induce a resistance response through the activation of defense pathways resulting in a more resistant phenotype. An alternative explanation for the observed resistance of plants exposed to damage-induced plant volatiles (DIPVs) is through the passive adsorption of defensive volatiles, thereby conferring volatile-mediated associational resistance [sensu (Himanen et al. 2010, 2015)], also referred to as environmentally acquired chemical camouflage (Kessler and Kalske 2018). Recent work has demonstrated that plants can adsorb exogenous VOCs and reemit them into the atmosphere (Niinemets et al. 2014; Li and Blande 2015) or sequester VOCs in their waxy cuticle (Camacho-Coronel et al. 2020; Mofikoya et al. 2020), leading to decreased herbivory (Li and Blande 2015; Mofikoya et al. 2020), disease (Camacho-Coronel et al. 2020) and disruption of host-location by parasitoids (Bui et al. 2021). The stronger resistance response after exposure to damaged kin that share the same chemotype could be explained by dose-dependent response by herbivores to passively acquired repellent or toxic VOCs. Receiver plants exposed to DIPVs from emitter plants of the same chemotype may acquire higher concentrations of chemotype-associated compounds compared to receiver plants exposed to DIPVs from plants of different chemotypes. Indeed, several DIPVs used to assign plants to chemotypes in sagebrush repel arthropods, including camphor (Obeng-Ofori et al. 1998; Mesbah et al. 2006), α-thujone (Tampe et al. 2015), and artemisia ketone (Liu et al. 2021). Thus, a sufficient test of the KSH must include a means of distinguishing between the active process of volatile-mediated induced resistance (VMIR) and the passive process of volatile-mediated associational resistance (VMAR).

Here we first tested the hypothesis that undamaged sagebrush plants (receivers) exposed to volatiles from damaged plants (emitters) will exhibit a stronger resistance response compared to when receiver plants are exposed to filtered air independent of chemotype. We then tested the importance of chemotype on the strength of the resistance response through two exposure experiments of all possible combinations of emitter and receiver plants using five predetermined chemotypes. Three plant responses were assessed: (1) herbivory using a choice-feeding assay with a generalist herbivore, (2) gene expression of a panel of genes known to be up-regulated when exposed to DIPVs, and (3) induced VOC emissions of intact plants. As demonstrated previously in the field but with only two chemotypes (Karban et al. 2014a), we expected to see a stronger resistance response when plants were exposed to the same chemotype as opposed to different chemotypes. The inclusion of the gene expression assay served to distinguish between an active (VMIR) and passive response (VMAR). We assumed that observable resistance in our feeding assays would be associated with up-regulation of defense-related genes if VMIR occurred, whereas no transcriptional changes would indicate that VMAR was the mechanism underpinning the reduction in herbivory. We further hypothesized that if receiver plants were exposed to emitter plants and adsorbed VOCs, (1) then the headspace of those receiver plants would contain VOCs associated with the chemotype of the emitter plant and (2) higher concentrations of the VOCs that are produced by the receiver plant that are shared with the emitter plant of the same chemotype.

## Materials and Methods

### Plant Propagation and Chemotype Identification

Sagebrush plants (*Artemisia tridentata* ssp. *vaseyana*) were grown from seed in a glasshouse at the University of Eastern Finland, Kuopio, Finland. Seeds were collected in the fall of 2018 from multiple populations across the Sierra Nevada Mountain Range as well as from the USGS seedbank (ESM, Table 1) and sown in 0.8 L plastic pots containing a mix of peat, soil, and sand (3:1:2). Plants were transferred to environmental growth chambers seven days prior to the initiation of exposure experiments.

**Table 1:** Results from GLMMs from two experiments, separately and combined, where the preference and amount of chewing damage was recorded from two bioassays with different emission treatments (damage-induced plant volatiles (DIPVs) vs. filtered air (FA); DIPVs exposed: same vs. different chemotype) with *C. morosus* in Petri dish arenas. Four (α-thujone, artemisia ketone, β-thujone, and camphor) and two chemotypes (artemiseole and α-thujone) were used in the first and second experiment, respectively. ET and RC represent emission treatment and receiving chemotype, respectively. Bold text indicates P values ≤ 0.1

Bioassay treatments		Experiment 1			Experiment 2			Combined		
		*X* ^*2*^	df	P	*X* ^*2*^	df	P	*X* ^*2*^	df	P
Preference										
DIPVs vs. FA	ET	2.52	1	0.11	0.65	1	0.42	3.02	1	**0.08**
	RC	0.27	3	0.96	0.00	1	1.00	0.19	4	1.00
	ET x RC	4.44	3	0.22	0.39	1	0.53	4.05	4	0.40
DIPVs: same vs. different	ET	0.62	1	0.43	2.72	1	**0.10**	0.05	1	0.83
	RC	3.66	3	0.31	0.00	1	1.00	3.89	4	0.42
	ET x RC	1.47	3	0.69	0.00	1	1.00	3.86	4	0.43
Leaf damage										
DIPVs vs. FA	ET	1.47	1	0.23	1.00	1	0.31	2.56	1	0.11
	RC	1.99	3	0.57	0.43	1	0.51	2.51	4	0.64
	ET x RC	2.52	3	0.47	0.04	1	0.80	2.08	4	0.72
DIPVs: same vs. different	ET	2.02	1	0.15	4.34	1	**0.04**	0.11	1	0.73
	RC	8.45	3	**0.04**	0.01	1	0.91	9.45	4	**0.05**
	ET x RC	3.16	3	0.36	0.00	1	0.95	7.59	4	0.11

To determine the chemotype of each sagebrush plant, we used direct headspace sampling of constitutively emitted volatiles from 5 leaves as described elsewhere (Grof-Tisza et al. 2021). The software ‘MSD ChemStation’ was used to identify the compounds by comparing mass spectra and retention times to published databases (NIST11, NIST, USA; WILEY275 mass spectral library; Wiley, Palo Alto, CA, USA). Chemotype assignment was based on motifs of discriminating dominant compounds in the overall emission blend as described previously (Karban et al. 2016; Grof-Tisza et al. 2021).

### Exposure System and Experimental Design

**Fig. 1:**
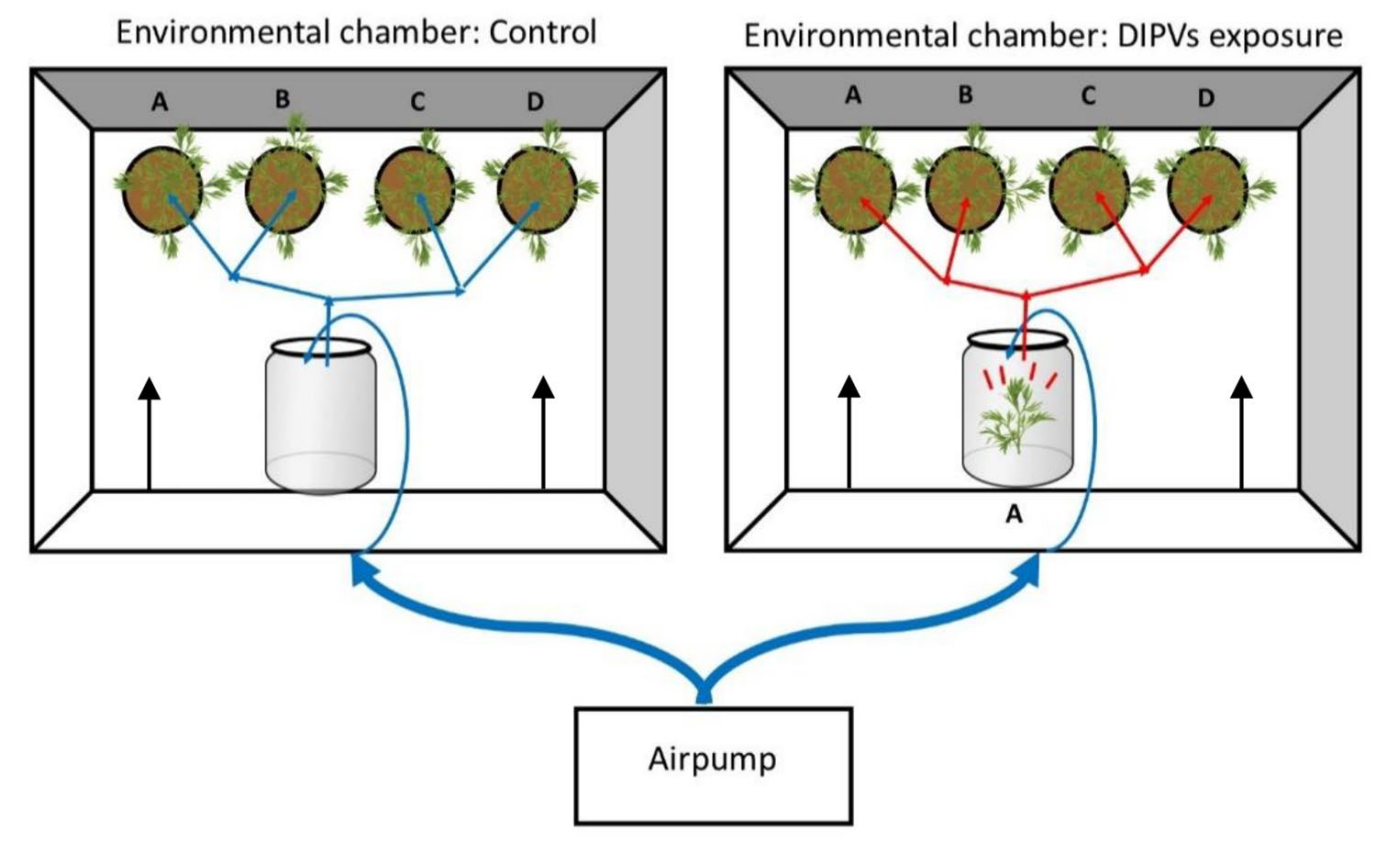
A conceptual diagram of the DIPVs exposure system. In the experimental chamber, a damaged emitter branch of chemotype A (emission source of DIPVs) was present in a glass mixing chamber, while the mixing chamber remained empty in the control chamber. An external air pump was used to create airflow into the mixing chamber and then to undamaged receiver plants of chemotypes A-D. Unidirectional airflow of the experimental chamber as indicated by the black arrows reduced movement of volatiles between receiver plants. Using this design, receiver plants used in all subsequent experiments were exposed to equal amounts of activated carbon-filtered air (blue line) or air containing DIPVs (red line)

Paired volatile exposure experiments were conducted in four controlled environment chambers (Weiss Technik, Lindenstruth, Germany) with horizontal laminar flow. In all chambers, activated carbon-filtered air was pumped through Teflon tubes into a 1 L glass jar (mixing chamber) at a flowrate of 1 L ml/min^− 1^. Outlet air from the mixing chamber was split twice leading to 4 receiver sagebrush plants, each receiving equal airflow (250 ml/min^− 1^; Fig. 1). In two of the controlled environment chambers, we placed one damaged emitter branch, 6–8 cm in length inside the mixing chamber. Twenty leaves on the emitter branch were damaged by making cuts perpendicular to the central vein. This damaged branch served as the emission source of DIPVs in the experimental treatment. The mixing chambers inside the two other controlled environment chambers remained empty such that receiver plants only received filtered air to provide a control treatment. The duration of the experimental exposure was 24 h. Receiver plants were not enclosed due to potential issues with condensation. The positioning of receiver plants (~ 20 cm apart and < 10 cm from exhaust vent) decreased the likelihood of VOC exposure from adjacent treatments. The environmental conditions in the chambers simulated those of the sagebrush growing season (early summer) with an artificial light-dark cycle (14 L: 10D), day-night temperature (26˚C :10˚C); and day-night relative humidity (60%:80%).

Differential germination and seedling survivorship produced an uneven number of plants representing each chemotype. As a result, we conducted two separate paired exposure experiments. The first experiment used 81 plants representing four chemotypes (α-thujone, artemisia ketone, β-thujone, and camphor) and the second experiment used 53 plants representing two chemotypes (α-thujone, artemiseole). All combinations of emitter and receiver chemotypes were assessed (ESM, Table 2). Upon completion of the exposure experiment, headspace volatiles of each receiver plant were collected using dynamic headspace sampling (Karban et al. 2014a) to assess induced volatile emissions; subsequently, leaves were collected for immediate use in behavioural bioassays or frozen in liquid nitrogen for gene expression analysis. While not a focus of this study, chemotypes of seedlings in our common garden reflected those of the source populations (ESM, Table 1), providing further evidence that chemotypes are non-plastic, heritable traits independent of edaphic conditions.

**Table 2:** Results of permutational multivariate analysis of variance on emission rates of all compounds combined and ecologically important subsets, green leaf volatiles (GLVs) monoterpenes and sesquiterpenes, for two exposure experiments as well as when combined. Four (α-thujone, artemisia ketone, β-thujone, and camphor) and two chemotypes (artemiseole and α-thujone) were used in the first and second exposure experiment, respectively. ET and RC represent emission treatment (filtered air n = 42; versus DIPVs, n = 45) and receiving chemotype, respectively. Bold text indicates P values ≤ 0.1

Response	Factor	Experiment 1			Experiment 2			Combined		
		Df	F	P	Df	F	P	Df	F	P
All compounds	ET	1	1.45	0.29	1	2.41	**0.05**	1	0.86	0.51
	RC	3	13.89	**< 0.01**	1	9.22	**< 0.01**	4	17.11	**< 0.01**
	ET x RC	3	1.11	0.35	1	0.88	0.475	4	1.49	**0.09**
GLVs	ET	1	0.45	0.66	1	0.27	0.77	1	0.48	0.64
	RC	3	6.83	**< 0.01**	1	0.33	0.72	4	8.17	**< 0.01**
	ET x RC	3	1.18	0.29	1	1.23	0.131	4	1.08	0.38
Monoterpenes	ET	1	1.20	0.27	1	2.66	**0.05**	1	0.48	0.79
	RC	3	17.3	**< 0.01**	1	11.82	**< 0.01**	4	17.12	**< 0.01**
	ET x RC	3	1.26	0.24	1	0.60	0.64	4	1.48	**0.10**
Sesquiterpenes	ET	1	2.28	**0.07**	1	0.36	0.85	1	1.71	0.16
	RC	3	7.22	**< 0.01**	1	0.93	0.41	4	5.28	**< 0.01**
	ET x RC	3	1.49	0.17	1	1.02	0.38	4	1.21	0.27

### Herbivore Bioassays

Behavioural choice tests with a generalist herbivore, the Indian walking stick (*Carausius morosus*), were conducted to assess the resistance of sagebrush leaves after the exposure experiments. Insects were reared in our lab-maintained colony at UEF and fed on a diet of brassicaceous plants. All insects were starved for 24 h prior to the experiment. Preliminary experiments suggested no underlying preference for any chemotype (data not shown).

**Fig. 2:**
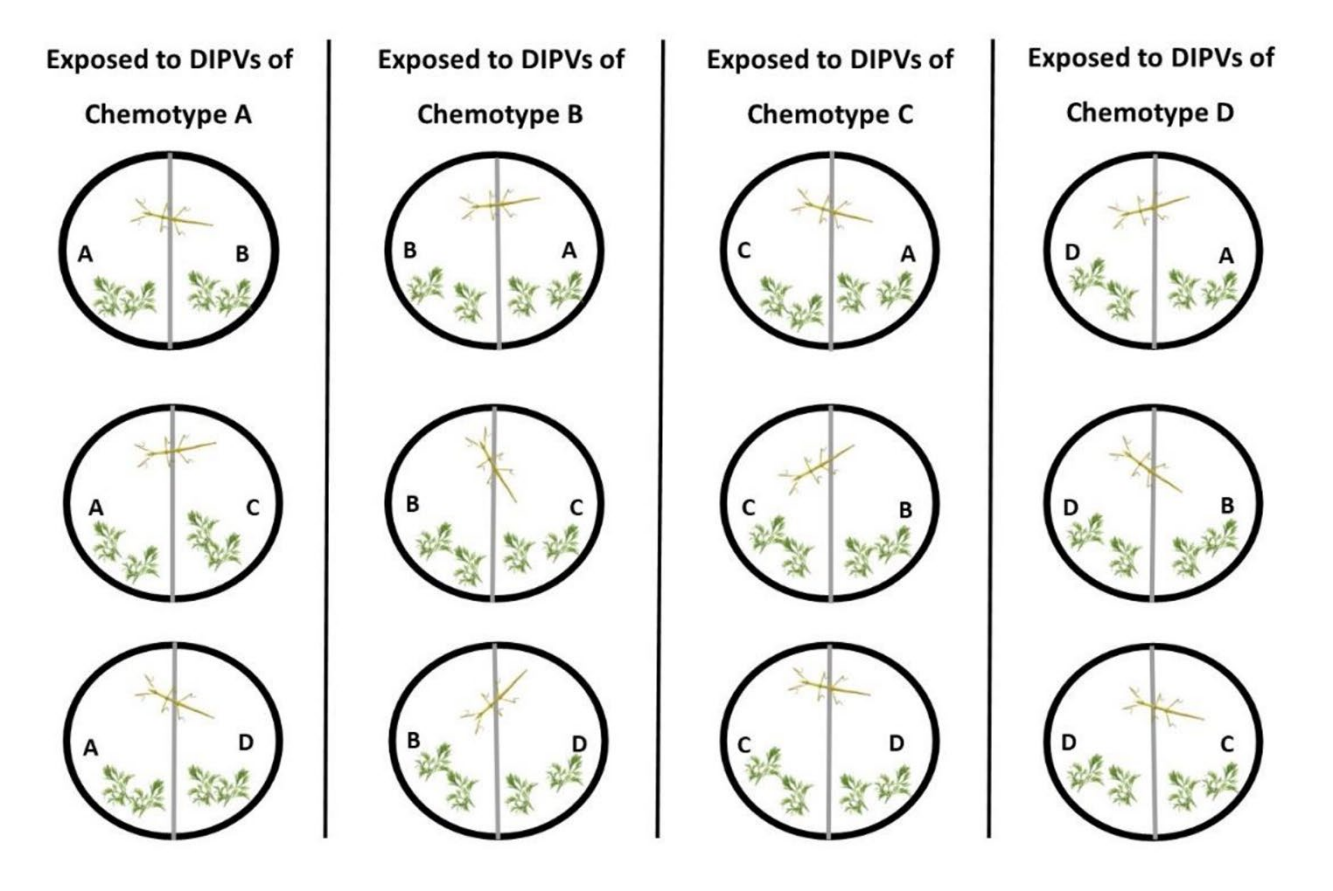
A conceptual diagram of feeding bioassay involving exposure to same versus different DIPVs. In each Petri dish arena, we placed 2 leaves from 2 plants. One set of leaves was from a receiver plant with the same chemotype (A) as the emitter branch (A), while the other set of leaves was exposed to the same DIPVs but from a plant with a different chemotype (B) than the emitter branch (A). One *C. morosus* individual that had previously been starved for 24 h was added to each arena. All possible combinations were compared

Preference for plants exposed to DIPVs from an emitter branch or filtered air (FA) was determined by placing two leaves from a DIPVs-exposed and FA-exposed plant of the same chemotype on opposite sides of a Petri dish arena along with one *C. morosus* individual. By restricting our comparisons between treatments of leaves of plants sharing the same chemotype, we controlled for any underlying differences among chemotypes. After 24 h of feeding, leaf damage was visually estimated. This was duplicated for each experimental plant with leaf location within the arena being switched between replicate trials. These resulting data were analyzed in two different ways. First, leaf damage was converted to a binary response (‘preferred, not preferred’) by categorizing the leaf pair with more damage as ‘preferred’. Secondly, we assessed the leaf area consumed independent of the paired design. These same metrics were used to assess herbivore preference for leaves exposed to DIPVs from the same or different chemotypes. All combinations of chemotypes were tested as a volatile source (emitter) and as a receiver plant (Fig. 2).

### Gene Expression

Primers were developed using published genomes of *A. tridentata* and *A. annua* for five genes previously found to be differentially up-regulated in lima bean (*Phaseolus lunatus*) after exposure to HIPVs (Arimura et al. 2000). These genes encode the pathogenesis-related protein, β-1,3-glucanase (PR-2); lipoxygenase (LOX-1 and LOX-2); phenylalanine ammonia-lyase (PAL); and farnesyl pyrophosphate synthetase (FPS). Gene expression was determined by quantitative real-time PCR (qRT-PCR). 50 mg of frozen sagebrush leaves from a subset of plants was disrupted using a TissueLyser II (Qiagen, Venlo, Netherlands) with precooled tube adapters. Total RNA was isolated using the NucleoSpin RNA Plant kit (Macherey-Nagel, Düren, Germany) and RNA quality was assessed using a nanodrop spectrophotometer (Thermo Fischer Scientific Waltham, MA, USA). RNA was reverse transcribed with the VERSO cDNA kit (Thermo Fischer Scientific). The qRT-PCR assay was performed on the LightCycer instrument (Roche, Rotkreuz, Switzerland) using the 480 SYBR Green Master Mix (Thermo Fischer Scientific). The relative gene expression levels of the target genes were calculated using the 2 – ΔΔCt (Wong and Medrano 2005) and base 2 log-transformed (Quackenbush 2002). The *Artemisia annua* actin gene was used as an internal standard. Primer sets and PCR conditions are provided in an online supplement (ESM Table 3).

**Table 3:** Mean (± 1 SE) of VOC emission (ng g^− 1^ h^− 1^) and results from linear mixed effect models to test for passively acquired compounds

Focal chemotype & compound	Receivers exposed to focal VS other emitter chemotypes				Focal receivers exposed to focal emitter VS filtered air (FA)			
	VOC emission	n	Z	P	VOC emission	n	Z	P
Artemisia ketone	8.9 ± 2.13	72 (other)	2.09	**0.04**	4529.60 ± 1532.67	7 (FA)	3.51	**0.01**
	47.05 ± 31.94	6 (focal)			15,747 ± 4526.00	2 (focal)		
α-Thujone	52.45 ± 19.53	50 (other)	-1.25	0.21	1862.22 ± 827.41	16 (FA)	0.42	0.69
	5.82 ± 1.81	10 (focal)			597.09 ± 353.01	5 (focal)		
Artemiseole	90.98 ± 38.27	70 (other)	0.34	0.74	46.27.98 ± 11.05	8 (FA)	-0.76	0.46
	147.43 ± 139.57	8 (focal)			234.47 ± 222.46	5 (focal)		
β-Thujone	144.70 ± 45.27	73 (other)	-1.20	0.23	876.38 ± 281.60	7 (FA)	0.42	0.69
	61.41 ± 45.89	6 (focal)			677.41 ± 181.97	2 (focal)		
Camphor	172.44 ± 46.07	71 (other)	-0.09	0.93	1078.82 ± 1023.26	7 (FA)	0.01	1.00
	103.1 ± 49.15	6 (focal)			235.75.1 ± 72.92	2 (focal)		

### Induced VOC Emission and Passive Adsorption

To assess induced VOC emission and passive adsorption, we used dynamic headspace sampling coupled with gas chromatography-mass spectrometry (GC-MS) analysis. A subset of receiver plants (n = 92) from all chemotypes and exposure treatments (i.e., filtered air and DIPVs) were enclosed in a 35 × 43 cm plastic bag (Polyethylene terephthalate; Look® Isopussi Eskimo oy, Finland; pre-heated at 120 °C for 1 h) that was fastened to the stem with a twisty tie. Activated carbon-filtered air was pumped into the bag for 5 min at a flowrate of 800 ml/min^− 1^. After this initial flushing to displace any VOCs that were present, the flowrate of the inflowing filtered air was reduced to 400 ml/min^− 1^. During collection, headspace volatiles were drawn out of the bag at a flow rate of 200 ml/min^− 1^ and collected in Tenax TA-filled stainless-steel tubes with 200 mg absorbent for 10 min.

Samples were analyzed by GC-MS (Hewlett Packard GC type 6890, Waldbronn, Germany; MSD 5973, UK). Trapped compounds were desorbed with an automated thermal desorption unit (Perkin Elmer ATD400 Automatic Thermal Desorption System, Wellesley, MA, USA) at 250 °C for 10 min and cryofocused at − 30 °C. The compounds were then transferred in a splitless mode to an HP-5MS capillary column (0.25 μm×60 m×0.25 μm, Agilent Technology, USA). The carrier gas was helium. Oven temperature was held at 40 °C for 2 min, and then programmed to ramp by 5 °C.min − 1 to 210 °C, and then by 20 °C.min − 1 to 250 °C under a constant flow of 1.2 mL min − 1. Compound identification was made by comparison with analytical standards (Sigma-Aldrich) using the software MSD ChemStation. For the unknown compounds, we calculated the retention indices (RI), through the injection of alkanes C8-C20 and compared their mass spectra to those in the NIST and Wiley libraries. Compound quantification was based on using the Total Ion Chromatograms (TIC) and according to the responses of analytical standards. VOC emission rates (ER) were calculated (Eq. 1) and expressed as (ng, hr^− 1^, g^− 1^).

1 $$ER=\frac{\left(Area\; of\; compound*Flowrate\; into\; the\; bag \right(L/min)}{\left(Dry\; biomass\; \right(g\left)*Time \right(hr\left)*Flowrate\; out\; of\; the\; bag\; \right(L/min)}$$

### Statistical Analysis

We used generalized linear mixed models (GLMMs) to assess the effect of emission source (DIPVs, filtered air, and individual chemotypes comprising the DIPVs group) and the chemotype of receiving plants on leaf preference and leaf area consumed (glmmTMB; Brooks et al. 2017). To assess the effects of specific emitter and receiver chemotypes and their interactions, we analyzed the data for the two exposure experiments separately as they contained different chemotypes (ESM Table 2). We also combined both exposure experiments to increase statistical power to investigate main effects of emission treatment (‘filtered air versus ‘DIPVs’ and ‘DIPVs from ‘same’ versus ‘different’ chemotypes). Binomial and Gaussian error distributions were used when modeling leaf preference and leaf area consumed, respectively. Petri dish arena identity nested within trial date as well as plant identity were used as random intercepts. Because the control and experimental treatments were imposed in separate replicate chambers, we assessed the effect of chamber identity using the same model structure used in the bioassay analyses to ensure no systematic errors were present. Model fit and subsequently the importance of each parameter, was assessed using likelihood ratio tests. Linear mixed models were used to assess the effect of emission source and gene on log_2_ fold expression change. Plant identity and trial date were used as random intercepts. Model assumptions and overdispersion were evaluated for each model when appropriate. All figures were constructed using raw data.

Induced VOC emission profiles of each receiver plant were subjected to non-metric dimensional scaling (NMDS) and perMANOVA using the vegan package (v2.5.6 ; Oksanen et al. 2019). The Bray-Curtis dissimilarity calculation was used with the fewest dimensions (k = 5) to achieve the best fit. These analyses were conducted for all compounds together and separately for ecologically important classes, monoterpenes, green-leaf volatiles, and sesquiterpenes. Upon detecting a group effect, the effect of the exposure treatment on individual compound emissions was assessed using GLMMs. The same random effect structure was used as described above and chemotype was included as a fixed effect. Compounds were transformed to meet model assumptions.

We assessed passive adsorption and reemission of chemotype-defining compounds (α-thujone, β-thujone, artemiseole, artemisia ketone, and camphor) in two ways. First, emissions of a focal compound were compared between receiver plants either exposed to emitter plants of the chemotype associated with the focal compound or all other chemotypes. Receiver plants of the same chemotype as the emitter plant were excluded from the analysis as their expression of the focal compound was expected. We hypothesized that chemotype-associated compounds emitted by plants of a particular chemotype would be detectable in the headspace of receiver plants generally not associated with these compounds if they were passively adsorbing volatiles. For example, we might expect receiver plants of chemotypes other than the α-thujone chemotype to emit α-thujone after being exposed to α-thujone emitter plants. Second, the emission of a compound associated with a focal chemotype was compared between receiver plants of the focal chemotype that were either exposed to emitter plants of the focal chemotype or filtered air. We hypothesized that plants of a focal chemotype should be associated with increased emission of the chemotype-associated compound if exposed to emitter plants of the focal chemotype. For example, we might expect α-thujone receiver plants to be associated with increased emission of α-thujone after exposure to α-thujone emitter plants as compared to when exposed to filtered air. The same model structure as described for individual compound emissions was used to address these questions. Upon detection of evidence supporting the adsorption and reemission of a chemotype-associated compound, transcription activity for receiver plants exposed to the emitter chemotype associated with the adsorbed compound would be assessed and compared to when exposed to filtered air using the same model structure as described above. All statistical tests were conducted in R (version R-4.0.3; R Development Core Team 2020).

## Results

### Choice-Test Bioassays

**Fig. 3:**
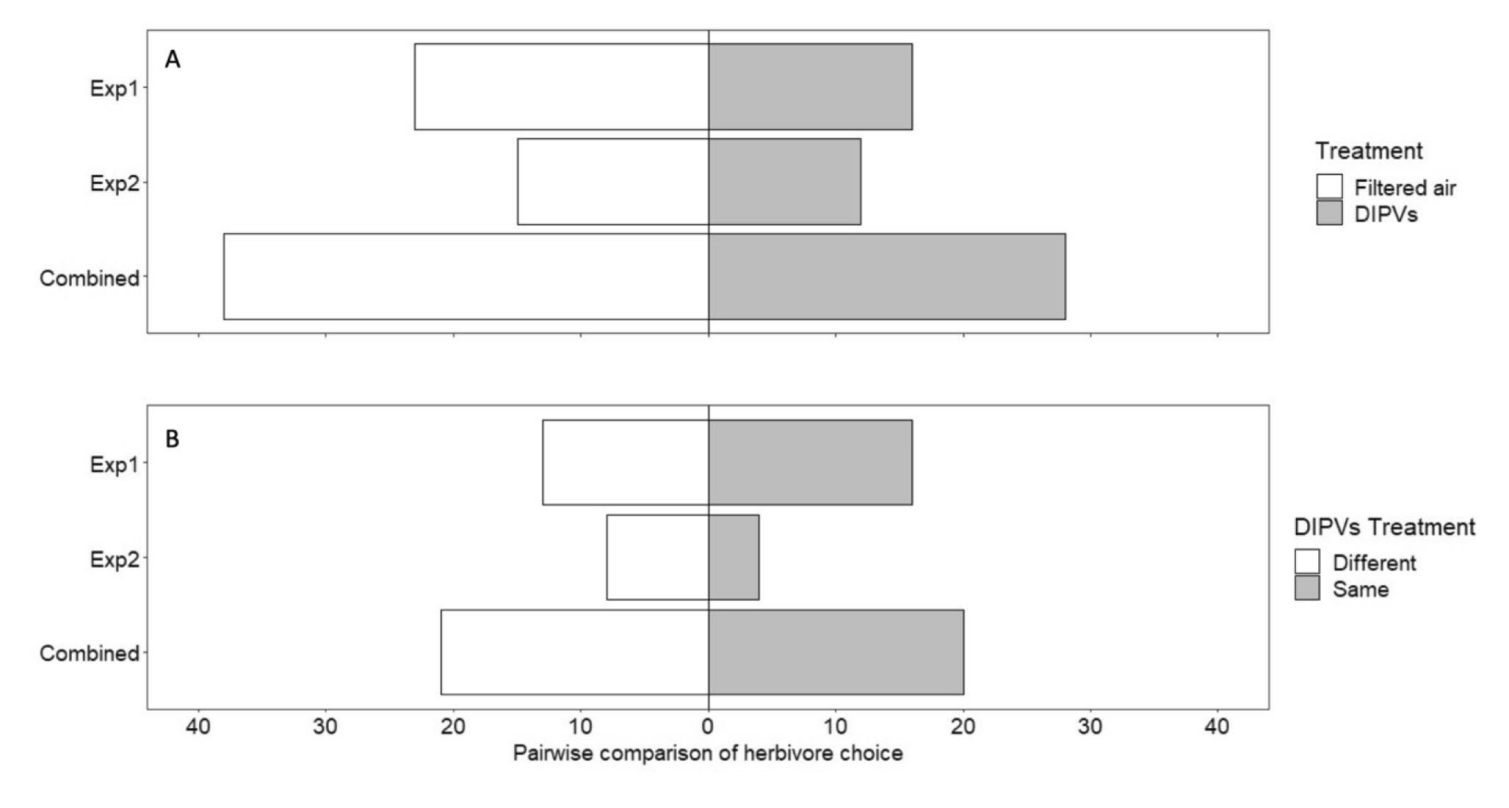
Preference of *C. morosus* in Petri dish arenas for leaves exposed to either (A) DIPVs or filtered air and (B) DIPVs from damaged leaves from plants of the same or a different chemotype. Four (artemisia ketone, α-thujone, β-thujone, and camphor) and two chemotypes (α-thujone and artemiseole) were used in the first and second experiment, respectively, with the total number of preferences represented by the combined plots. No differences among chemotypes were detected

**Fig. 4:**
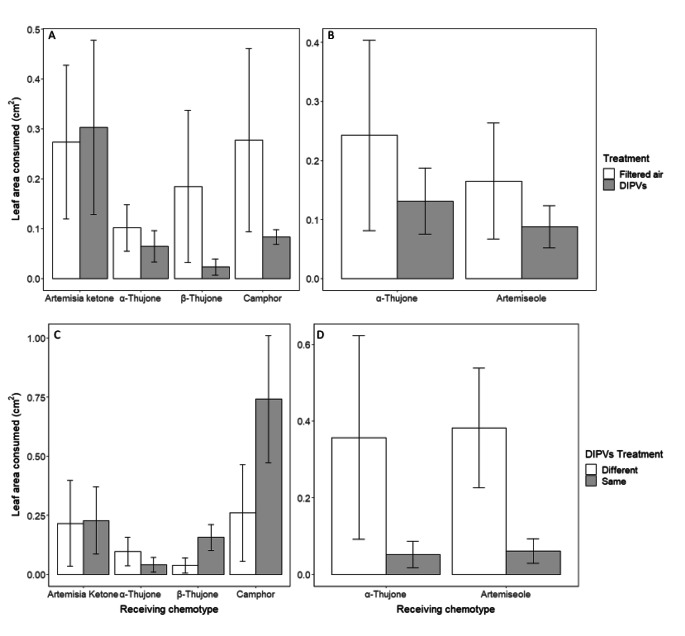
Mean (± 1 SE) leaf area consumed of leaves exposed to different treatments by *C. morosus* in Petri dish areas. In A-B, leaves from different plants of the same chemotype were either exposed to DIPVs or filtered air (FA) in two exposure experiments using (A) 4 and (B) 2 chemotypes, respectively (n of trials per chemotype by treatment (DIPVs, FA); experiment 1: Artemisia ketone 12,12; α-thujone 9,10; β-thujone 7,5; camphor 10,12; experiment 2: α-thujone 10,10; artemiseole 17,17). In C-D, leaves from different plants were either exposed to DIPVs of the same or different chemotype in two exposure experiments using (C) 4 and (D) 2 chemotypes, respectively (n of trials per chemotype by DIPVs treatment (different, same) for each experiment, experiment 1: Artemisia ketone 9,9; α-thujone 8,10; β-thujone 6,7; camphor 6,9; experiment 2: artemiseole, 6,6; α-thujone 6,6

Leaves exposed to DIPVs became marginally more resistant to herbivory compared to leaves exposed to filtered air. In total for both exposure experiments when comparing the responses of plants exposed to filtered air or DIPVs, no leaf damage was observed in 41% of trials (46/112), and these were omitted from the analysis. In nearly 58% of the remaining trials (38/66), control leaves were preferred over DIPV exposed leaves (28/66; Fig. 3a; Table 1). This finding was significant when using a one-tailed test in agreement with our directional hypothesis based on previous findings stating that plants exposed to wounding signals would have less damage compared to controls (Z =-1.71, P = 0.04). Control leaves had 1.7x more damage than DIPV exposed leaves (Fig. 4a-b; Table 1). No differences among receiving chemotypes were detected.

Compared to the trials comparing plant responses when exposed to filtered air or DIPVs, a higher proportion of trials (49%; 40 out of 81) were omitted because of no observed feeding damage when comparing responses of plants exposed to DIPVs from emitter plants of the same or different chemotype. This increase of omitted trials was not statistically different (*X*^*2*^ = 0.76, df = 1, P = 0.17). Results were mixed between the two exposure experiments. In 55% (16/29) of trials involving 4 chemotypes, herbivore individuals preferred leaves that were exposed to DIPVs from the same chemotype over those exposed to a different chemotype (Fig. 3b; Table 1) and these leaves had 2.2x more damage (Fig. 4c). This result is largely driven by a single trial with camphor, where a leaf pair exposed to DIPVs received an unusually high level of damage. Because of the number of omitted trials, we did not have sufficient replication to test interactive effects between receiving and emitting chemotypes (ESM Table 4). In the second exposure experiment with 2 chemotypes, individuals preferred leaves exposed to different chemotypes in 66% (8/12) of the trials (Fig. 3b) and control leaves had 5.6x more damage compared to those exposed to DIPVs (Fig. 4d). Moreover, we found a significant interaction between the receiving and emitting chemotype (Z = 4.55, P = 0.03). Detecting this interactive effect was expected given that we detected a significant effect of emission treatment (same vs. different chemotypes) and only two chemotypes were used. The results for the second experiment indicated that for the α-thujone and artemiseole plants, exposure to the same chemotype resulted in a stronger resistance response than exposure to DIPVs from a different chemotype.

### Gene Expression

**Fig. 5:**
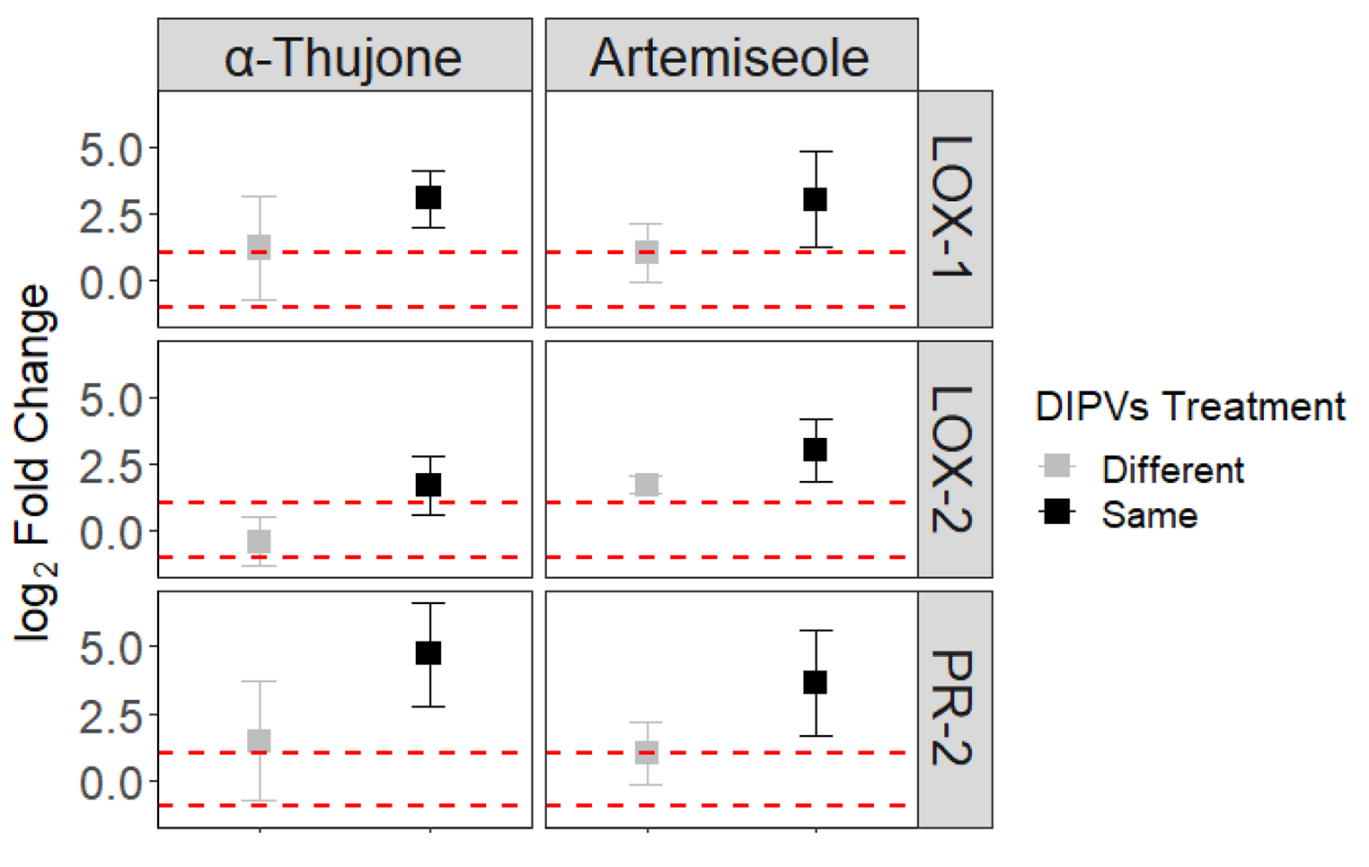
Mean (± 1 SE) of log2 fold-change of three genes from receiver plants chemotypes exposed to DIPVs from plants of the same or different chemotype. The upper and lower horizontal dotted lines 100% increase or decrease of expression, respectively

Exposure to DIPVs induced the expression of 3 of the 5 defense genes tested, LOX-1, LOX-2, PR-2 (ESM Fig. 1). FPS and PAL were not found to be differentially regulated and were omitted from further analysis. Plants exposed to DIPVs (n = 29) were associated with moderately increased gene expression of all genes with an aggregate Log_2_ fold-change (LFC) of 2.5 (SE ± 0.36) compared to plants exposed to filtered air (n = 9; LFC mean ± SE, 0.28 ± 0.33; ESM Fig. 2). This increase was significant if testing our *a priori* directional hypothesis stating an expectation of defensive gene upregulation in response to DIPVs (T = -1.615, P = 0.05). Receiving chemotype had a strong effect on gene expression (ESM Fig. 1, *X*^2^ 14.97, DF = 4, P < 0.01). The effect of emitting chemotype was weaker (*X*^2^ = 6.94, DF = 4, P = 0.14). With the data from both exposure experiments combined, we did not detect a difference between plants exposed to the same (n = 12) or different (n = 17) chemotypes (*X*^2^ = 0.01, DF = 1, P = 0.93). The quality of many of our RNA extractions was poor, potentially from the high phenolic content of sagebrush (Loomis 1974) and consequently were omitted. Due to insufficient replication of representative chemotypes, we were unable to assess interactive effects of emitting and receiving chemotype pairs for all possible combinations with the experiments combined or the first experiment alone. We did detect a difference between plants exposed to DIPVs from the same or different chemotypes in the second exposure experiment with just two chemotypes α-thujone (n = 4) and artemiseole (n = 6) (Fig. 5). While all genes were up-regulated in both treatments (‘same’ and ‘different’), plants exposed to DIPVs of the same chemotype showed higher levels of gene transcription. Individual genes were not significantly different between the two treatments, likely due to the small sample size. However, the aggregate gene response was significant (*X*^2^ = 4.14, DF = 1 P = 0.04); the aggregate mean LFC of plants exposed to DIPVs from the same chemotype was 2.7x higher than those exposed to DIPVs from different chemotypes. This finding is in alignment with the herbivore bioassay: leaf pairs from receiver plants of the α-thujone and artemiseole chemotypes experienced less herbivore damage when exposed to DIPVs from emitter plants of the same chemotype compared to DIPVs from plants of a different chemotype.

### Induced VOC Emissions and Passive Adsorption

**Fig. 6:**
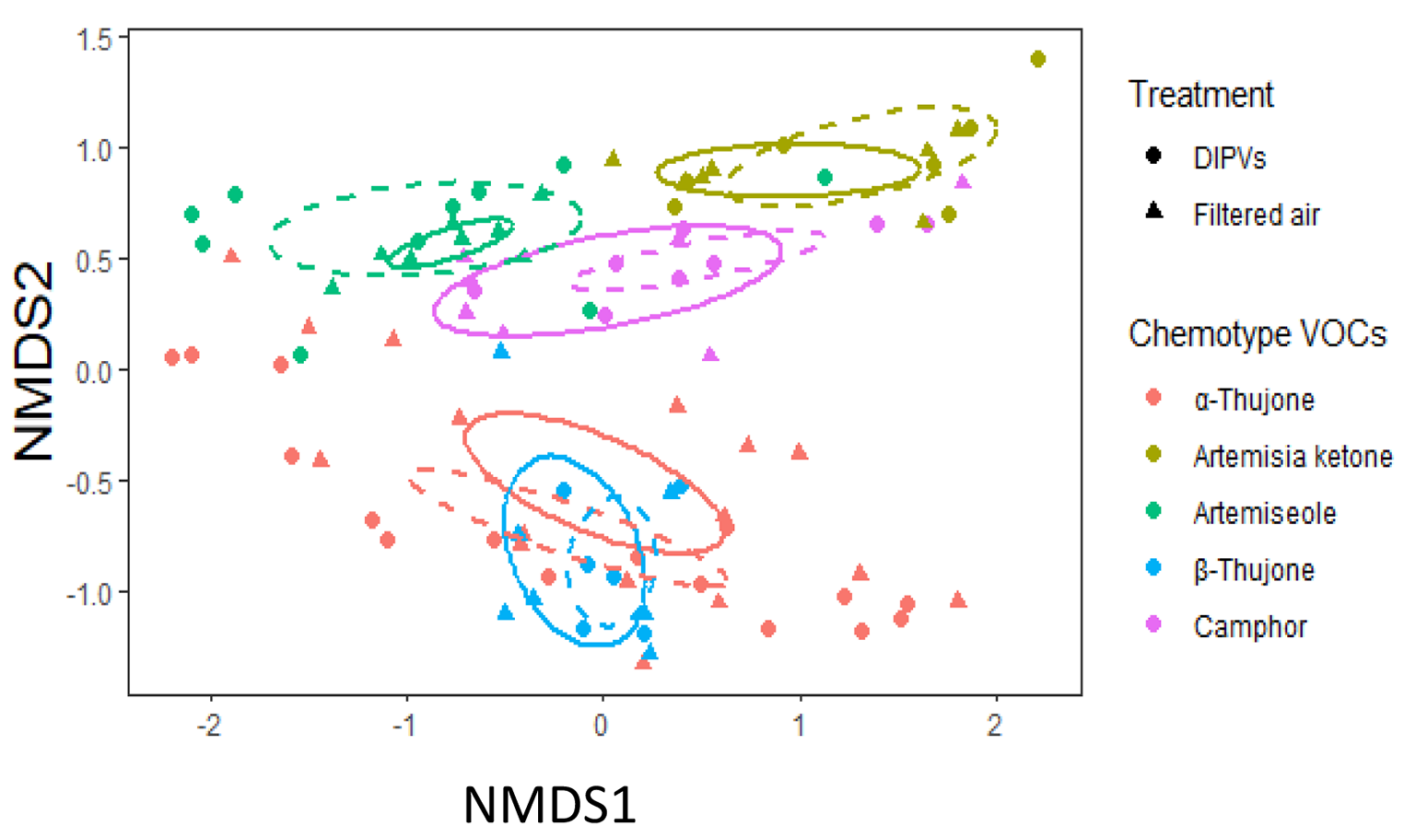
NMDS plot of total induced VOC emissions of undamaged plants of 5 different chemotypes exposed to damage-induced plant volatiles or filtered air. Ellipses represent a 95% confidence interval around the centroid for each chemotype. The dashed and solid ellipses represent the control exposed treatments, respectively (DIPVs, Filtered air: artemisia ketone 7,7; α-thujone 16,16; β-thujone 6,7; artemiseole, 10,8; α-thujone 6,6; camphor 8,7)

Emission profiles of intact plants were clearly separated by chemotypes (Fig. 6; Table 2, ESM Table 5). This multivariate analysis validates our assignment of chemotypes based on motifs of dominant compounds. When comparing induced emissions between plants exposed to DIPVs or filtered air, we found marginally insignificant differences among sesquiterpenes in exposure experiment 1 and among all compounds and monoterpenes for exposure experiment 2. Of the sesquiterpenes, the emissions of β-caryophyllene and bicyclogermacrene, increased 1.8x and 2.4x, respectively (ESM, Table 6). Among the monoterpenes in exposure experiment 2, the emissions of artemisia triene increased, while that of α-phellandrene decreased 3.4x. Several individual monoterpenes and oxygenated monoterpenes were found to show changes in induced emissions, including profile dominating compounds used in chemotype assignment such as α-thujone and artemisia ketone; the emission of these compounds increased 1.8x and 1.7x, respectively. No difference was detected between plants exposed to the same or different chemotypes with data from both exposure experiments combined (ESM Table 7).

**Fig. 7:**
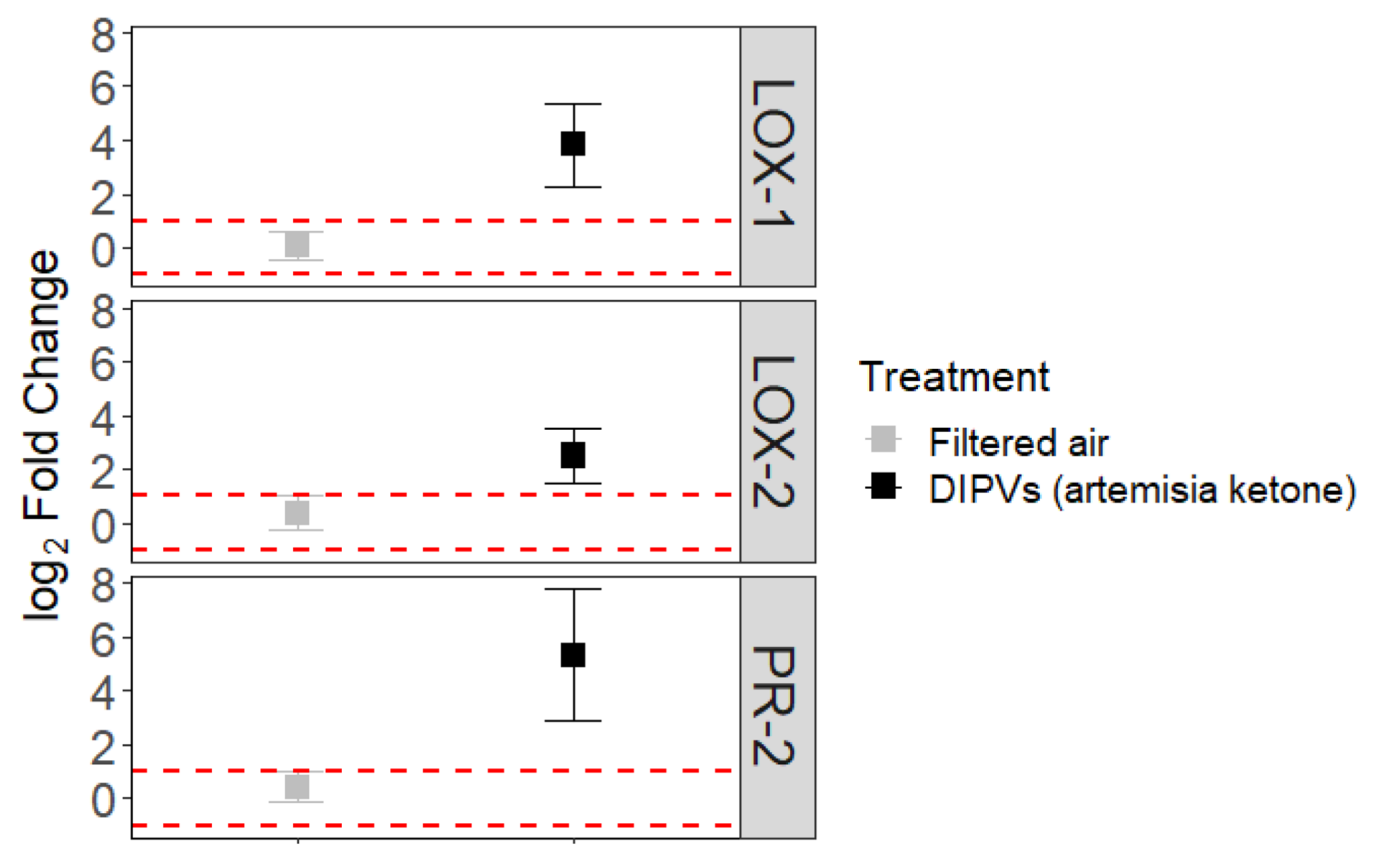
Mean (± 1 SE) of log2 fold-change of three genes from receiver plants exposed to filtered air (n receiver plants per chemotype: artemisia ketone, 3; α-thujone, 2; β-thujone, 1; 2 artemiseole, 2; camphor, 1) or DIPVs (n receiver plants per chemotype; artemisia ketone, 2; α-thujone, 1; β-thujone, 2; camphor, 3) from emitter plants of the artemisia ketone chemotype. The upper and lower horizontal dotted lines 100% increase or decrease of expression, respectively

We found evidence of passive adsorption of VOCs (Table 3). Receiver plants (excluding those of the artemisia ketone chemotype) exposed to emitter plants of the artemisia ketone chemotype emitted 5.2x more artemisia ketone than those exposed to DIPVs from chemotypes other than artemisia ketone or filtered air. Moreover, artemisia ketone receiver plants emitted 3.4x more artemisia ketone after exposure to artemisia ketone emitter plants compared to those exposed to filtered air. This suggests that during the 24 h exposure, receiver plants adsorbed artemisia ketone and this compound was subsequently emitted into the headspace of the plant, potentially conferring associational resistance. Indeed, a total of 14 out of 21 (binomial probability, P = 0.06) *C. morosus* individuals preferred leaves exposed to filtered air and DIPVs from different chemotypes than when exposed to DIPVs from plants of the artemisia ketone chemotype. Exposure of receiver plants to DIPVs from artemisia ketone plants was associated with an increase in gene expression relative to when exposed to filtered air (LOX-1, Z=-2.54, P = 0.01; LOX-2, Z= -1.92, P = 0.05; PR-2, Z=-2.49, P = 0.01; Fig. 7).

## Discussion

The kin selection hypothesis (KSH) has been invoked in several studies to explain the observation of decreased herbivory in plants exposed to VOCs emitted from damaged plants that are more genetically related compared to those that are less related (Karban et al. 2013, 2014a; Moreira et al. 2016; Hussain et al. 2019). Many of these studies neglected to consider a simpler alternative hypothesis of volatile-mediated associational resistance (VMAR), where repellent or toxic VOCs are adsorbed on neighbouring plants rendering these intact plants more resistant to herbivores (Himanen et al. 2010, 2015; Kessler and Kalske 2018). Our results demonstrated that sagebrush plants actively responded to VOC cues from damaged plants. Moreover, stronger volatile-mediated induced resistance (VMIR) was elicited when emitter and receiver plants shared the same heritable chemotype but only for two of the five chemotypes tested. We detected the ability of sagebrush plants to adsorb and subsequently release repellent VOCs. Consequently, both VMIR and VMAR may be responsible for observed decreases in herbivory after exposure to damaged plants depending on the chemotypes of receiver and emitter plants involved.

In alignment with numerous studies from several researchers that have repeatedly demonstrated the ability of sagebrush plants to respond to wounding cues from damaged neighbors [e.g., (Karban et al. 2006; Pezzola et al. 2017; McMunn 2017; Grof-Tisza et al. 2020)], we found that receiver plants exposed to DIPVs experienced less herbivory by a generalist herbivore compared to control plants. More notably, we showed for the first time that this reduction in herbivory was associated with the up-regulation of defense-related genes. Exposure to DIPVs can prime plants without transcriptional changes (Engelberth et al. 2004; Heil 2014). Consequently, not detecting a response at the gene-level is not sufficient evidence to conclude that plants did not perceive DIPVs. Conversely, finding an increase in gene expression is a strong indicator that plants actively perceived and responded to VOC cues. Our ability to detect direct induction through the up-regulation of genes enabled us to distinguish between active and potentially passive responses. These results validated our approach to investigating volatile-mediated interactions under laboratory conditions in a system historically studied using manipulative field studies.

While it is well established that plants can respond to DIPVs emitted by nearby plants (Karban et al. 2006; Kost and Heil 2006; Li and Blande 2017), how the traits of emitter and receiver plants and their interactive effects influence the ability of receiver plants to respond to these cues is less understood. Several investigations reported that the degree to which a plant responded depended on its relatedness to the damaged emitter (Karban et al. 2013; Moreira et al. 2016; Hussain et al. 2019). A few of these studies specifically assessed the role of heritable chemotypes in volatile-mediated signalling (Karban et al. 2014a; Hussain et al. 2019). For example, Karban et al. (2014a) concluded that communication was more effective between sagebrush plants sharing the same chemotype relative to when the chemotypes were different. Based on this finding, the authors posited that kin selection could explain the selection for ‘private-channels of communication’ between related individuals. These authors, like many of those listed above, assumed plants actively responded to DIPVs, but never confirmed this assumption by quantifying physiological or transcriptional changes. This additional verification is crucial to distinguish between active and passive responses as these same observations are possible via VMAR. Here, we obtained the equivalent result as first described by Karban and colleagues (2014) in our second exposure experiment with two chemotypes, α-thujone, and artemiseole; exposure of a receiver plant to DIPVs from an emitter plant of the same chemotype resulted in less herbivory compared to that from a different chemotype. Unlike the original study, we obtained evidence indicative of an active response. No evidence was detected to suggest that the decrease in herbivory was a function of adsorbed VOCs. Taken together, these results confirm the conclusions of previous work and lend additional support for the KSH.

As mentioned above, a previous field study with sagebrush investigated volatile-mediated interactions between two chemotypes, a-thujone and camphor (Karban et al. 2014b). Due to a low sample size resulting from lack of herbivory in our feeding assays, we could not rigorously assess this combination of chemotypes in our first exposure experiment with four chemotypes which included the two originally tested. The pairing of chemotypes in our second exposure experiment involving α-thujone and artemiseole was chemically similar to that of α-thujone and camphor. The volatile blend of camphor resembles that of artemiseole (Fig. 6) (Grof-Tisza et al. 2021). One hypothesis that may explain this parallel pattern of induction between studies involving plants of similar chemotypes is that the chemical dissimilarity between α-thujone and the camphor dominant chemotypes of camphor and artemiseole prevent the cross recognition of volatile cues. In ordination space, α-thujone clusters quite distinctly from artemiseole and camphor (Fig. 6). This hypothesis is supported by one model explaining the evolution of plant-to-plant communication, which is thought to have evolved as a by-product of within-plant signalling (Heil and Karban 2010). Selection may reinforce the specificity of chemotype-specific alarm cues as the ability to respond to specific cues and minimize eavesdropping confers a competitive advantage. Numerous studies have demonstrated the high level of specificity of volatile cues involved in plant signalling (Erb et al. 2015; Moreira et al. 2018; Ninkovic et al. 2021).

Plants can adsorb and reemit toxic or repellent VOCs that confer associational resistance to insects (Himanen et al. 2010, 2015; Mofikoya et al. 2020), pathogens (Camacho-Coronel et al. 2020) and can alter the cues used by natural enemies to locate their hosts (Bui et al. 2021). We detected artemisia ketone in the headspace of receiver plants generally not associated with this volatile compound suggesting it was adsorbed and reemitted. Additionally, we detected higher concentrations of this VOC in the headspace of receiver plants of the artemisia ketone chemotype after exposure to damaged emitter plants of the same chemotype. Because artemisia ketone is known to repel insects (Liu et al. 2021), it is plausible that receiver plants might have benefited from the increased protection provided by the adsorbed VOC in addition to its own emission. This could explain the observed preference by *C. morosus* for leaves other than those exposed to artemisia ketone emitter plants; if herbivores respond to adsorbed VOCs in a dose-dependent fashion, exposure to DIPVs of a plant sharing the same chemotype would result in less damage. This observation might lead to the erroneous conclusion that plant-to-plant signalling is more effective between like chemotypes when it is a result of VMAR. Here, we found that plants exposed to artemisia ketone were associated with increased transcription of all genes tested relative to control plants suggesting that the reduced herbivory was at least in part a function of VMIR. It is conceivable that both VMAR and VMIR contributed to decreased consumption of artemisia ketone exposed leaves. However, we are unable to determine the relative importance of each in this study. We did not detect adsorption of the other chemotype-dominant compounds tested, α-thujone, camphor, artemiseole, and β-thujone. Studies investigating interspecific VMAR benefited from the presence of uniquely expressed VOCs, enabling researchers to easily track deposited VOCs (Bui et al. 2021). Contrastingly, the VOCs assessed here were not uniquely emitted by each chemotype, and their emission rates varied substantially even within the same chemotype. Considering this variation, differentiating between adsorption and primary emission is not easily accomplished. It is possible these VOCs contributed to VMAR to some extent.

Several studies have demonstrated threshold effects in plants in response to stress [reviewed in (Niinemets et al. 2014)]. For example, Karl et al. (2008) found that under moderate levels of thermal stress, volatile phytohormones and induced VOCs were absent or were detectable at low levels. Above a particular threshold, LOX products and methyl salicylate increased substantially. Though largely untested, theory predicts the selection for threshold-mediated responses to volatile alarm cues as a means to conserve resources for more substantial or immediate threats (Orrock et al. 2015). Indeed, a mechanistic explanation of plant priming is that threshold levels of stressors that trigger the activation of plant defense are decreased when plants are primed (Morrell and Kessler 2014). VMAR in conjunction with threshold-mediated induced resistance provides an alternative explanation to that of plants differentially responding to cues from kin and strangers. The accumulation of adsorbed VOCs on a plant emitted by damaged neighbours of a similar chemotype could trigger transcriptional changes upon reaching a critical threshold of a particular VOC cue. While the effect of exposure to a chemically similar individual is the same as VMIR, the selective driver of this effect may not involve kin recognition. To our knowledge, such a mechanism has not been described.

We detected minor differences in the emissions of several VOCs across multiple functional classes between exposed plants and filtered air. The emission of several of these compounds are known to be inducible and exhibit repellent properties, reduce damage by herbivores, or aid in indirect defenses. For example, β-caryophyllene emission was increased in intact maize after exposure to wounding signals (Engelberth et al. 2004); it was shown to repel a psyllid pest using *Arabidopsis* over-expression and knock-out lines (Alquézar et al. 2017) as well as attract natural enemies of *Spodoptera* caterpillars (Köllner et al. 2008). A few of these differentially emitted VOCs were used in chemotype assignment, including α-thujone, artemisia ketone, and artemisia triene, (Grof-Tisza et al. 2021) and are known to function as direct defenses (Obeng-Ofori et al. 1998; Mesbah et al. 2006; Tampe et al. 2015; Liu et al. 2021). Direct induced emissions are often less pronounced than the emissions seen following secondary damage after previous VOC exposure (i.e., priming) [reviewed in (Frost et al. 2008)]. Mechanically damaging receiver plants prior to the collection of headspace VOCs may have yielded larger effects although this was not tested here. It is possible that the observed increased emissions for at least some of these VOCs may stem from the adsorption and reemission and not direct induction. Given the growing evidence of VMAR, researchers should exercise caution when interpreting VOC emissions of plants exposed to DIPVs of strongly aromatic plants like sagebrush.

### Conclusion

The seminal studies with sagebrush which first suggested that plants exhibit kin recognition assumed plants were actively responding to the VOC cues of related individuals but did not test the mechanism of resistance. A similar result was achievable through the adsorption of repellent or toxic VOCs. Our results confirmed the conclusions of these previous studies by demonstrating that volatile-mediated induced resistance is chemotype-dependent. Adding to this previous work, we showed that sagebrush could adsorb and reemit repellent VOCs potentially contributing to volatile-mediated associational resistance. The relative benefits of these mechanisms remain untested.

## Electronic Supplementary Material

Below is the link to the electronic supplementary material.

Supplementary Material 1

## Data Availability

Data is available upon request.

## Code Availability

Code is available upon request.
