# Supplementary material for: Volatile-Mediated Induced and Passively Acquired Resistance in Sagebrush (Artemisia tridentata)
Source: J Chem Ecol. 2022 Aug 19;48(9-10):730–45. doi: 10.1007/s10886-022-01378-y (PMC9618528; doi:10.1007/s10886-022-01378-y)
Supplement: Supplementary file 1 — Supplementary Material 1 [file 10886_2022_1378_MOESM1_ESM.docx]

Electronic Supplementary Material

Table 1. Source of sagebrush seeds

| Accession #/Pop. | Source | State | GPS Coordinates |
| --- | --- | --- | --- |
| W6-47587 | USGS | WY |  |
| W6-35296 | USGS | WY |  |
| W6-47985 | USGS | CA |  |
| Mt. Rose | Self-collected | NV | 39.18, 119.92 |
| SNARL | Self-collected | CA | 37.61, 118.83 |
| Sagehen | Self-collected | CA | 39.43, 120.24 |
| Monitor Pass | Self-collected | CA | 38.68, 119.59 |
| Spooner Summit | Self-collected | CA | 39.1, 119.9 |

Table 2. Number of independent replicate plants for each chemotype used in two exposure experiments where receiver plants were exposed to activated-carbon filtered air (control) or an emitter branch that was used as a source of DIPVs.

|  | Experiment 1 | | |  | Experiment 2 | | |
| --- | --- | --- | --- | --- | --- | --- | --- |
|  |  | Treatment | |  |  | Treatment | |
|  |  | DIPVs | Control |  |  | DIPVs | Control |
| Emitter chemotype | α-Thujone | 2 |  |  | α-Thujone | 3 |  |
|  | Artemisia ketone | 3 |  |  | Artemiseole | 3 |  |
|  | β-Thujone | 2 |  |  |  |  |  |
|  | Camphor | 2 |  |  |  |  |  |
|  |  |  |  |  |  |  |  |
| Receiver chemotype | α-Thujone | 11 | 10 |  | α-Thujone | 10 | 11 |
|  | Artemisia ketone | 9 | 9 |  | Artemiseole | 14 | 12 |
|  | β-Thujone | 7 | 8 |  |  |  |  |
|  | Camphor | 9 | 9 |  |  |  |  |

Table 3: Primer sequences used for the RT-PCR analysis. All primers were derived from *Artemisia annua* except for FPS and PAL, which were derived from *A. tridentata.*

| **Gene and ID** | **Forward primer** | **Reverse primer** |
| --- | --- | --- |
| β-Actin reference EU531837 (Olofsson et al, 2011) | CCCCTGCTATGTATGTTGCCA | CGCTCGGTAAGGATCTTCATCA |
| FPS (Farnesyl pyrophosphate synthase) KJ609177 | CTCATACACGCAGAGGGCAA | CGTTGAACAGGTCCACGAGA |
| PAL (Phenylalanine ammonia lyase)  JX489399.1 | AAGCAGTCAGAGTCGCGTTT | TCGCCCGTCAAGTAAACTCC |
| PR2 (β-1-3-glucanase) PWA60789.1  CTI12_AA379430 | ACCTTCCGAACAAGATGTGG | CTGGATCGTTGAGGGATTGT |
| LOX-1 Lipase/lipoxygenase PWA92286.1, CTI12_AA082020 | GGCATCAGATGGTGGTGTGA | ACAGCAGGCGATTGAGGAAA |
| LOX-2Lipase/lipoxygenase |  |  |
| PWA78264.1, CTI12_AA192480 | CCTTCCTGCCGACCTAATC | TTCTTGTCTCCATGCCCCAC |

**Revere-transcription PCR conditions:** 42°C for 30 minutes, 94°C for 2 min, 4° C
**PCR conditions:** 95 for 10 sec for 1 cycle followed by 95°C for 20 s, 55°C for 20 s, and 72°C for 20 s for 45 cycles, with a final extension at 72°C for 5 min.  Melting curve analysis was performed after PCR.

Olofsson L, Engström A, Lundgren A, Brodelius PE. Relative expression of genes of terpene metabolism in different tissues of Artemisia annua L. BMC Plant Biol. 2011;11:45. doi: 10.1186/1471-2229-11-45

Table 4. Remaining possible combinations of emitter and receiving chemotypes after removal of trials where no herbivory was recorded. Lack of replication prevented the ability to rigorously investigate interactive effects between emitting and receiving chemotypes.

| Emitter Chemotype | Receiving Chemotype | Replicates |
| --- | --- | --- |
| Artemisia Ketone | Artemisia Ketone | 3 |
| Artemisia Ketone | α-Thujone | 4 |
| Artemisia Ketone | β-Thujone | 3 |
| Artemisia Ketone | Camphor | 2 |
| α-Thujone | Artemisia Ketone | 3 |
| α-Thujone | α-Thujone | 1 |
| α-Thujone | β-Thujone | 1 |
| α-Thujone | Camphor | 3 |
| β-Thujone | Artemisia Ketone | 2 |
| β-Thujone | α-Thujone | 2 |
| β-Thujone | β-Thujone | 1 |
| β-Thujone | Camphor | 4 |
| Camphor | Artemisia Ketone | 4 |
| Camphor | α-Thujone | 3 |
| Camphor | Camphor | 3 |
| Camphor | β-Thujone | 0 |

Table 5. Results from a pairwise permutational ANOVA to compared receiver chemotypes

| Pairwise-test | | F | P value |
| --- | --- | --- | --- |
| α-Thujone | Artemisia ketone | 20.98 | 0.002 |
| α-Thujone | Artemiseole | 7.06 | 0.008 |
| α-Thujone | β-Thujone | 4.55 | 0.020 |
| α-Thujone | Camphor | 6.07 | 0.001 |
| Artemisia ketone | Artemiseole | 20 | 0.001 |
| Artemisia ketone | β-Thujone | 14.49 | 0.001 |
| Artemisia ketone | Camphor | 14.13 | 0.001 |
| Artemiseole | β-Thujone | 18.92 | 0.001 |
| Artemiseole | Camphor | 3.63 | 0.030 |
| β-Thujone | Camphor | 4.48 | 0.001 |

Table 6. Mean (±SE) VOC emission rate (ng g^-1^ h^-1^) of plants after 24h exposure to filtered air or damage-induced plant volatiles (DIPVs). P values from negative binomial GLMM. Bolded letters indicate P values < 0.1


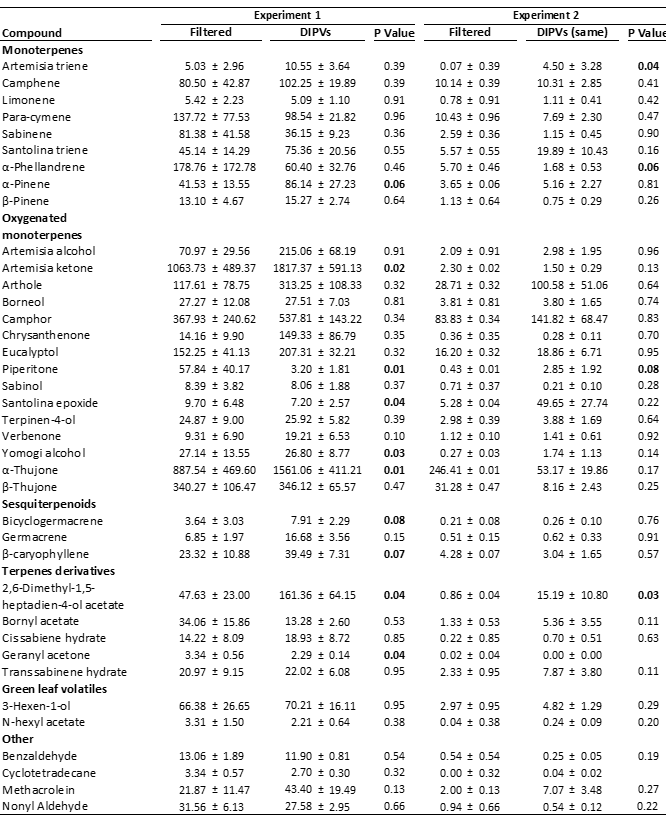


Table 2. Results of permutational multivariate analysis of variance on emission rates of all compounds combined and ecologically important subsets, green leaf volatiles (GLVs) monoterpenes and sesquiterpenes, for two exposure experiments as well as when combined. 4 (α-thujone, artemisia ketone, β-thujone, and camphor) and 2 chemotypes (artemiseole and α-thujone) were used in the first and second exposure experiment, respectively. ET and RC represent emission treatment (same n=15 versus different n=27 DIPVs) and receiving chemotype, respectively. Bolded text indicates P values ≤ 0.1.

| Response |  | Factor |  | Experiment 1 | | |  | Experiment 2 | | |  | Combined | | |
| --- | --- | --- | --- | --- | --- | --- | --- | --- | --- | --- | --- | --- | --- | --- |
|  |  |  |  | Df | F | P |  | Df | F | P |  | Df | F | P |
| All compounds |  | RC |  | 3 | 9.5 | **<0.01** |  | 1 | 3.71 | **<0.01** |  | 4 | 8.11 | **<0.01** |
|  |  | ET |  | 1 | 1.51 | 0.15 |  | 1 | 0.71 | 0.65 |  | 1 | 1.56 | 0.11 |
|  |  | RC X T |  | 3 | 1.46 | **0.08** |  | 1 | 0.77 | 0.61 |  | 4 | 1.23 | 0.18 |
|  |  |  |  |  |  |  |  |  |  |  |  |  |  |  |
| GLVs |  | RC |  | 3 | 9.73 | **<0.01** |  | 1 | 1.42 | 0.23 |  | 4 | 4.73 | **<0.01** |
|  |  | ET |  | 1 | 1.32 | 0.24 |  | 1 | 0.02 | 0.98 |  | 1 | 1.33 | 0.27 |
|  |  | RC X T |  | 3 | 2.05 | **0.09** |  | 1 | 0.71 | 0.50 |  | 4 | 0.48 | 0.90 |
|  |  |  |  |  |  |  |  |  |  |  |  |  |  |  |
| Monoterpenes |  | RC |  | 3 | 11.61 | **<0.01** |  | 1 | **5.86** | **<0.01** |  | 4 | 10.67 | **<0.01** |
|  |  | ET |  | 1 | 0.2 | 0.96 |  | 1 | 1.03 | 0.34 |  | 1 | 0.14 | 0.98 |
|  |  | RC X T |  | 3 | 1.26 | 0.27 |  | 1 | 0.36 | 0.86 |  | 4 | 0.91 | 0.53 |
|  |  |  |  |  |  |  |  |  |  |  |  |  |  |  |
| Sesquiterpenes |  | RC |  | 3 | 9.78 | **<0.01** |  | 1 | 2.17 | 0.10 |  | 4 | 5.89 | **<0.01** |
|  |  | ET |  | 1 | 1.43 | 0.25 |  | 1 | 0.91 | 0.46 |  | 1 | 1.52 | 0.18 |
|  |  | RC X T |  | 3 | 1.95 | **0.09** |  | 1 | 1.50 | 0.20 |  | 4 | 1.45 | 0.17 |


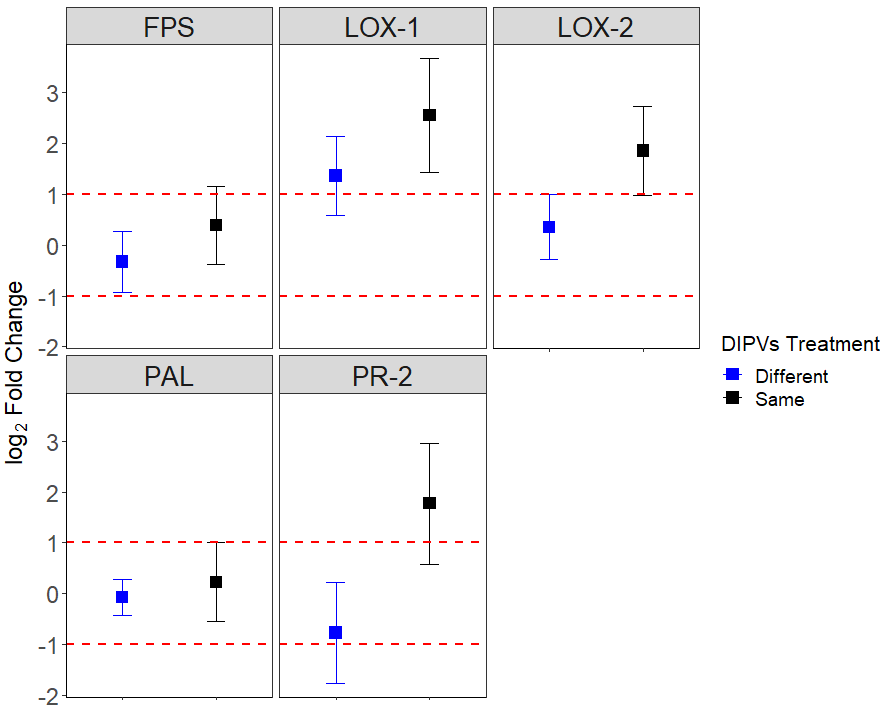


Figure 1. Results of gene expression analysis with 5 primer sets (FPS, LOX-1, LOX-2, PAL, and PR-2) using plants exposed to damaged-induced plant volatiles with the same (n= 5) or different (n=7) chemotypes as well as a filtered-air control =7). Two primer sets FPS and PAL did not responds to DIPVs (DIPVs exposed vs Filtered air) and were not used in subsequent trials (ANOVA::GLMM; FPS: *X^2^* = 0.0082 DF =1 ,P= 0.93, PAL: *X^2^* = 0.0057 DF=1, P= 0.94).


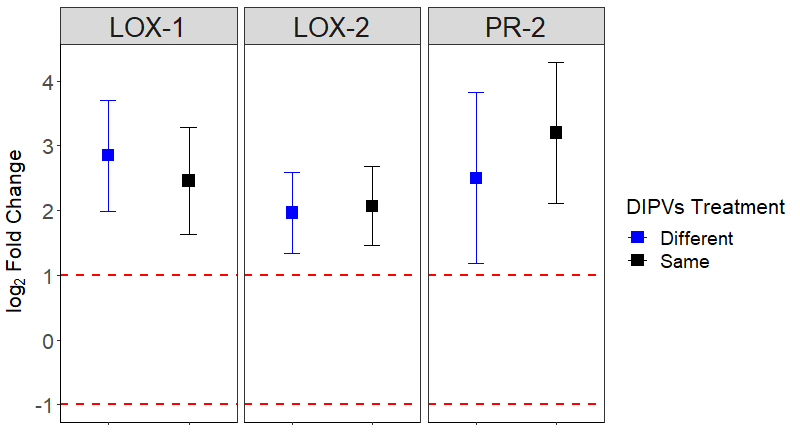


Figure 2. Aggregated log fold expression change of three genes (LOX-1, LOX-2, and PR-2) using plants exposed to damaged-induced plant volatiles with the same (n= 12) or different (n=17) chemotypes. No difference was detected (*X*^2^ = 0.01, DF=1, P= 0.93).
